# The effect of mitochondrial fusion on chondrogenic differentiation of cartilage progenitor/stem cells via Notch2 signal pathway

**DOI:** 10.1186/s13287-022-02758-7

**Published:** 2022-03-25

**Authors:** Safwat Adel Abdo Moqbel, Rong Zeng, Diana Ma, Langhai Xu, Changjian Lin, Yuzhe He, Chiyuan Ma, Kai Xu, Jisheng Ran, Lifeng Jiang, Lidong Wu

**Affiliations:** 1 Department of Orthopedic Surgery, The Second Affiliated Hospital, Zhejiang University, School of Medicine, Hangzhou, 310000 Zhejiang Province People’s Republic of China; 2 Orthopedic Research Institute of Zhejiang University, Hangzhou, Zhejiang Province People’s Republic of China; 3 Key Laboratory of Motor System Disease Research and Precision Therapy of Zhejiang Province, Hangzhou, Zhejiang Province People’s Republic of China; 4 Department of Pain, Zhejiang Provincial People’s Hospital, People’s Hospital of Hangzhou Medical College, Hangzhou, People’s Republic of China

**Keywords:** Osteoarthritis, Mitochondrial fusion, Cartilage progenitor**/**stem cell, *Mfn2*, *Notch2*

## Abstract

**Background:**

Osteoarthritis (OA) is a debilitating disease that inflicts intractable pain, a major problem that humanity faces, especially in aging populations. Stem cells have been used in the treatment of many chronic diseases, including OA. Cartilage progenitor/stem cells (CPSCs) are a type of stem cells with the ability to self- renew and differentiate. They hold a promising future for the understanding of the progression of OA and for its treatment. Previous studies have reported the relationship between mitochondrial dynamics and mesenchymal stem cell (MSC) proliferation, differentiation and aging. Mitochondrial dynamic and morphology change during stem cell differentiation.

**Methods:**

This study was performed to access the relationship between mitochondrial dynamics and chondrogenic differentiation of CPSCs. Mitochondrial fusion and fission levels were measured during the chondrogenic differentiation process of CPSCs. After that, we used mitochondrial fusion promoter to induce fusion in CPSCs and then the chondrogenic markers were measured. Transmission electron microscopy (TEM) and confocal microscopy were used to capture the mass and fusion status of mitochondria. Lentiviruses were used to detect the role of mitofusin 2 (*Mfn2*) in CPSC chondrogenic differentiation. In vivo, *Mfn2* was over-expressed in sheets of rat CPSCs, which were then injected intra-articularly into the knees of rats.

**Results:**

Mitochondrial fusion markers were upregulated during the chondrogenic induction process of CPSCs. The mass of mitochondria was higher in differentiated CPSC, and the fusion status was obvious relative to un-differentiated CPSC. Chondrogenesis of CPSCs was upregulated with the induction by mitochondrial fusion promoter. *Mfn2* over-expression significantly increased chondrocyte-specific gene expression and reversed OA through NOTCH2 signal pathway.

**Conclusions:**

Our study demonstrated that the mitochondrial fusion promotes chondrogenesis differentiation of CPSCs. *Mfn2* accelerates the chondrogenesis differentiation of CPSCs through *Notch2*. In vivo, *Mfn2*-OE in sheets of rCPSCs ameliorated OA in the rat model.

**Supplementary Information:**

The online version contains supplementary material available at 10.1186/s13287-022-02758-7.

## Introduction

Osteoarthritis (OA), the most common form of arthritis, spreads widely across aged populations and is characterized by loss of articular chondrocytes and joint failure [1]. Articular cartilage has a limited ability to self-repair, especially after trauma or degenerative disease due to its avascular character [2]. Nowadays, the treatment of OA is limited to symptomatic treatment.

Cartilage progenitor**/**stem cells (CPSCs) are a promising source of cells that possess the ability to differentiate into chondrogenic, osteogenic, and adipogenic tissue. Several studies have investigated the differential ability of CPSCs in vivo and in vitro*,* and they were able to survive in vivo after implantation [3–5]. Progenitor cells isolated from different tissues such as bone marrow, adipose tissue and umbilical cord hold potential and prospects for cartilage repair [6]. Furthermore, the intraarticular injection of stem cells in OA mice model has been shown to reduce inflammation and has chondroprotective effects [7, 8]. Additionally, the differentiation process of progenitor cells is associated with increasing mitochondrial mass.

Mitochondria is the most dynamic responsive system in the cell [9]. Mitochondrial dynamics, including fusion and fission, play a vital role in several biological processes in stem cells and are very important for the metabolic regulation of cellular energy [10]. Several studies have demonstrated the effect of mitochondrial dynamics during somatic cell differentiation [11]. Outer membrane fusion is mediated by mitofusins, *Mfn1* and *Mfn2*, while inner membrane fusion is controlled by Optic Atrophy 1 (*Opa1*) [12]. Previous studies showed that inhibiting fusion in progenitor cells leads to failure in differentiation [13]. Dynamic-related protein (DRP1) and fission-1 (*Fis1*) are the regulators of mitochondrial fission [14]. Inhibition of DRP1 by interfering RNA or Mdivi-1 increased cardiac differentiation of human pluripotent stem cells [15].

In light of these findings, we decided to study the mitochondrial dynamics of chondrogenic differentiation of CPSCs. We hypothesized that increasing cell mitochondrial fusion by over-expressing *Mfn2*, a key regulator of mitochondrial fusion, or using a chemical promoter, may promote chondrogenic differentiation of CPSCs. Mitochondrial fusion promoter M1 was employed to detect the role of mitochondrial fusion in chondrogenic differentiation of CPSCs, and then lentiviruses were used to detect the role of *Mfn2* in chondrogenic differentiation of CPSCs in vitro. A group of rats underwent medial meniscus resection and then received a sheet of rCPSCs with overexpressed *Mfn2* intra-articularly.

## Methods

### Materials and reagents

Fetal bovine serum (FBS), streptomycin, penicillin, 0.25% pancreatic enzyme and DMEM were obtained from Gibco, USA. Chondrogenic induction medium, osteogenic induction medium and adipogenic induction medium were obtained from Cyagen US Inc. Hematoxylin and BSA were obtained from Sigma-Aldrich, USA. Mitochondrial fusion promoter M1 was obtained from Selleckchem (Shanghai, China).

### Preparation of cartilage/stem progenitor Cells (CPSCs)

Cartilage was isolated from the knee and hip joints of twenty 3-week-old male Sprague–Dawley (SD) rats (Zhejiang Academy of Medical Science, Hangzhou China) as mentioned in [16]. In brief, the rats were euthanized with pentobarbital and then under sterile requirements the cartilages tissues were isolated. Cartilage tissues (≤ 1.5 g) were digested with 5 ml of 0.25% Trypsin–EDTA for 30 min. Afterward, the trypsin–EDTA solution was removed by centrifuge and then the tissues were washed with PBS. Next, the tissue samples were incubated with 6 ml DMEM containing 1 ml of 0.2% collagenase II for 4 h at 37 °C on a horizontal shaker. Subsequently, the cells were centrifuged and then suspended in DMEM containing 10% FBS, 100 U/ml penicillin and 100 µg/ml streptomycin. The cells were then seeded in tissue culture flasks at 37 °C with 5% CO_2_ as passage 0 (*P*0). When the cells in the culture flask reached a density above 80%, the cells were released into the medium with trypsin and collected to be reseeded. After passage 0, cells were seeded at very low density to form colonies (2–3 cells/cm^2^), and these cells were designated as CPSCs. The medium was replaced every 3 days, and after 10 days, the cell density reached over 80% in the culture flasks.

### Transduction of cells

In order to identify if mitochondrial fusion is able to affect CPSC differentiation, we chose *Mfn2* as a representative factor. To confirm the role of *Mfn2* during chondrogenic differentiation of CPSCs, endogenous *Mfn2* was over-expressed or down-regulated by lentiviral particles. According to its effectiveness, shRNA (sense: 5′ CCAAAUUGCUCAGGAAUAAATT-3′, anti-sense: 5′UUAUUCCUGAGCAAUUUGGTT-3′) was chosen to down-regulate the expression of *Mfn2*. A scrambled shRNA sequence (TTCTCCGAACGTGTCACGT) was used as a negative control group [*Mfn2*-KD control group (KD-NC)]. Lentivirus overexpression *Mfn2* (Lenti-*Mfn2*) particles and lentiviral GFP particles (lenti-control), Lentivirus- knockdown *Mfn2* particles (Lenti-KD), and knockdown control particles (ctrl-KD) were prepared by (Genechem; Shanghai; China). For infections, rCPSCs were incubated with lentiviral particles and Polybrene (5u/mL) in growth medium. After 24 h, the infection medium was replaced. GFP fluorescence was used to detect the transduction efficiency. The expression of *Mfn2* was measured by western blot and qRT-PCR. Lentiviral vectors were efficiently used to over-express or knock down *Mfn2* in > 80% of passage 3 (*P3)* rCPSCs.

### RNA Isolation and qRT-PCR assessment

CPSCs were cultured in a 6-well plate at a density of 10 × 10^4^cells/well. Total RNA was extracted using TRIzol® Plus RNA Purification Kit (Invitrogen; Thermo Fisher Scientific, Inc.) according to the manufacturer’s instructions. The ratio of the absorbance at 260 nm and 280 nm (A260/A280) was used to calculate and verify the quality and purity of the extracted RNA (200–300 ng/uL). After using PrimeScript RT Master Mix (Takara) to synthesize cDNA, qPCR was carried out on StepOnePlus Real-TimePCR system using SYBR Green qPCR SuperMix. mRNA expression was normalized using 18 s as a housekeeping gene. The complete list of primers used can be found in Table 1.

**Table 1:** Primer sequences used in this study

Gene	Accession number	Amplicon length (bp)	Forward	Reverse	Tm (°C)
Rat *Col2a1*	NM_012929.1	151	GGCCAGGATGCCCGAAAATTA	CCCTCTCTCCCTTGTCACCAC	61.5
Rat *Acan*	XM_039101035.1	196	CTGGGTGGATGCAGAGAGAC	TTGGTTTGGACGCCACTTCT	60.1
Rat *Sox-9*	NM_080403.2	170	AAGTCGGTGAAGAATGGGCA	GTCGGTGGACCCTGAGATTG	60.11
Rat *Mfn2*	NM_130894.4	118	TCAAGCGCCAGTTTGTGGAG	CACAGATGAGCAAATGTCCCAGA	60.17
Rat *Mfn1*	NM_138976.1	136	ATCTGGTGGAGATACAGGGCT	TCCCACAGCATTGCGTTGAT	60.61
Rat *Opa1*	NM_133585.3	108	GGCACTTCAAGGTCGTCTCA	CACTGCTCTTGGGTCCGATT	60
Rat *Drp1*	NM_053655.3	94	AGGTTGCCCGTGACAAATGA	CACAGGCATCAGCAAAGTCG	60.18
Rat *Fis1*	NM_001105919.1	108	ACGCCTGCCGTTACTTCTTC	GCAACCCTGCAATCCTTCAC	60.67
Rat *18S*	NR_046237.2	172	CCTGAGAAACGGCTACCACA	ACCAGACTTGCCCTCCAATG	60.96

### Protein isolation and western blot

Cells were seeded in a 6-well plate at a density of 10 × 10^4^ cells/well After treatment, cells were washed twice with phosphate-buffered saline (PBS). RIPA Lysis Buffer containing phosphatase inhibitor and protease inhibitor was added for 30 min to extract all proteins. The extracted protein was analyzed using a BCA quantification kit. Equivalent amounts of protein were separated on 8%-12% SDS-PAGE gels and transferred to polyvinylidene fluoride (PVDF) membranes. The PVDF membrane was blocked in TBST with 10% skim milk for 1 h and then incubated overnight with primary antibodies at 4 °C. Then, the PVDF membrane was incubated with the secondary antibodies for 1 h at room temperature. Blotting was performed using primary antibodies: Col2 (1:1000)(rabbit; no. ab34712; Abcam), GAPDH (1:1000) (rabbit; no. ab70699; Abcam), SOX-9 (1:1000) (rabbit; no. ab185966Ab; Abcam), Acan (1:1000) (rabbit; no. ab36861; Abcam), MFN2 (1:200) (mouse; no. Sc-100560; Santa Cruz) NICD (1:500) (rabbit; no. 10062-2-AP; Proteintech; Wuhan, China), MFN1(1:500) (rabbit; no. 13798-1-AP; Proteintech), NOTCH2 (1:1000) (rabbit; no. 5737; Cell signaling Tec.), HES1(1:1000) (rabbit; no. 11988; Cell signaling Tec), β-actin (1:200) (mouse; no. Sc-8432; Santa Cruz). Protein bands were visualized using an ECL kit and analyzed with Quantity One software. GAPDH and β-actin were used as controls in all western blot analysis.

### Safranin O and Alcian blue staining

The cells were cultured as a cell sheet in 12 well plate or pellet. and then treated according to the study design. According to manufacturer’s protocol, 3 × 10^5^ cells were centrifuged at 200*g* at 20 °C for 5 min, and then the pellet was cultured with 0.5 ml chondrogenic differentiation medium. The cells were washed three times with PBS and then fixed with 4% paraformaldehyde solution. Pellets were incubated in 30% sucrose (Sigma-Aldrich, MO, USA) at room temperature for 24 h, and then serial sections (8 µm) were prepared and stored at − 20 °C. Subsequently, 0.75% Safranin O (Sigma-Aldrich) or Alcian blue solution (1% in 3% acetic acid, pH 2.5; Cyagen) was used to stain the cells for 7 min at room temperature. After washing the cells three times with PBS, the images were captured using microscope or gross camera.

### Immunofluorescence staining

0.5% Triton X-100 was used to permeabilize the samples (pellets and knee joint sections) and then blocked with 5% BSA for 1 h. After that, the samples were incubated with primary antibodies for *Col2* (1:100)*, Mfn2* (1:50) *or Prg4* (1:50; Novus Biologicals) overnight at 4 °C. After 3 times of washing, the samples were then incubated with secondary anti-rabbit or anti-mouse antibodies (Alexa Fluor 488 or 555 Beyotime, China) for 1 h in the dark. Subsequently, the samples were counterstained with DAPI for 5 min and then washed with PBS and then visualized under fluorescence microscope.

### Mitochondrial dynamics imaging

rCPSCs were seeded on circle microscope cover glass in a 24-well plate at a density of 2.5 × 10^4^cells/well and then were cultured with or without chondrogenic induction medium (Chondrogenic basal medium, ITS supplement, TGF-β3, Sodium pyruvate, Ascorbate, Proline, and Dexamethasone). After 14 d of induction, the cells were incubated with Mito-Tracker Red (Beyotime, China) according to the manufacturer’s instructions. Fluorescence was captured using a Zeiss 510 Confocal microscope (Carl Zeiss, Germany).

### Transmission electron microscopy (TEM)

Cells were seeded in a 6-well plate at a density of 10 × 10^4^cells/well and then cultured with or without chondrogenic induction medium for 14 d. The cells were fixed with 2.5% glutaraldehyde 4 °C for 12 h. Using 2% osmium tetroxide to fix the samples for 1 h, the samples were dehydrated in an ascending series of acetone. After embedding, the samples were put in an oven to polymerize at 37 °C for 16 h and then at 60 °C for 14 h. The samples were wiped and then cut into semi-thin parts using ultramicrotome. Transmission electron microscope (Hitachi, Japan) was used to observe the cells.

### In vivo experiment

All the animals were obtained from Zhejiang Academy of Medical Science, Hangzhou China. In strict accordance with the instructions for the use and care of laboratory animals and with the approval of IACUC, a total of 40 male S-D rats (6-weeks old; 190–240 g) were used to establish the in vivo experiment. This study contains four groups: sham (negative control) group, OA (destabilization of the medial meniscus) group, rCPSC-OE (rCPSCs with *Mfn2-*OE) group, and rCPSC-OE-NC (rCPSCs with negative-control of *Mfn2*-OE) group (10 rats/group). Briefly, pentobarbital (40 mg/kg) was used to anesthetize the rats. Under sterile conditions, the knee joints were opened and then the medial meniscus was resected to induce OA in the rats. The rats in the sham group received sham surgery where the knee joints were cut open without resection of the medial meniscus. After surgery, each rat received an injection with penicillin (20000U/mL) and then divided randomly. The rCPSCs were isolated and cultured in cell flasks at 37 °C with 5% CO_2_ as mentioned above, and then rCPSC sheets were treated with *Mfn2*-OE or *Mfn2*-OE-NC and were passaged until *P*2. After that, the treated rCPSCs were collected and washed three times with PBS. After that, rCPSCs were prepared as cell suspensions of 1 × 10^6^/100µL per leg and were administered by intra-articular injection into the rCPSC-OE and rCPSC-OE-NC rats 2 weeks after surgery, whereas the rats in the sham and OA groups received equal amounts of PBS injection. The injected rCPSCs were allogeneic cells. The rats were killed eight weeks after surgery.

### Histological analysis

The rats were killed with intraperitoneal injection of (800 mg/kg) pentobarbital, and then, the knee joints were collected. The joints were first fixed in 4% paraformaldehyde and decalcified with 10% EDTA at room temperature for 2 months. The tissues were embedded in paraffin and cut into 5 μm sagittal sections. Slides of each joint were deparaffinized and rehydrated and then stained with Safranin O/fast green (S–O) and hematoxylin and eosin (H&E). The OARSI and Mankin score systems were used with three individuals for blinded histological evaluation.

### TUNEL staining

TUNEL staining was used to detect the degree of cartilage DNA damage in rats. Cartilage sections were deparaffinized, rehydrated and incubated with 0.1% Triton X-100 for 30 min and then stained with InSitu Cell Death Detection Kit according to the manufacturer’s instructions for 30 min at 37 °C. The nuclei were stained with DAPI, and the images were visualized using fluorescence microscope.

### Immunohistochemical analysis

Paraffin-embedded knee joint sections were prepared and then blocked with 5% BSA for 1 h. After that, the sections were incubated with primary antibodies against Col2 (1:100), SOX-9 (1:100), Acan (1:100), HES1 (1:100), MFN2 (1:50), MMP-3 (1:100) (rabbit; no. ab52921; Abcam), and MMP-13 (1:50) (rabbit; N3C1; GeneTex) at 4 °C for 12 h. Subsequently, the samples were washed and then incubated with secondary antibody (Boster Biological Technology) for 2 h. Optical microscope was used to capture the images.

### Statistical analysis

All the data were recorded more than 3 times, and statistical differences were analyzed using SPSS software (version 22.0; IBM, USA). All data are presented as mean ± standard deviation (SD). One-way analysis of variance (ANOVA) followed by Tukey’s post hoc test was used to assess the statistical difference between the groups. *P* less than 0.05 was considered as significant difference.

## Results

### Identification of CPSCs

**Fig. 1:**
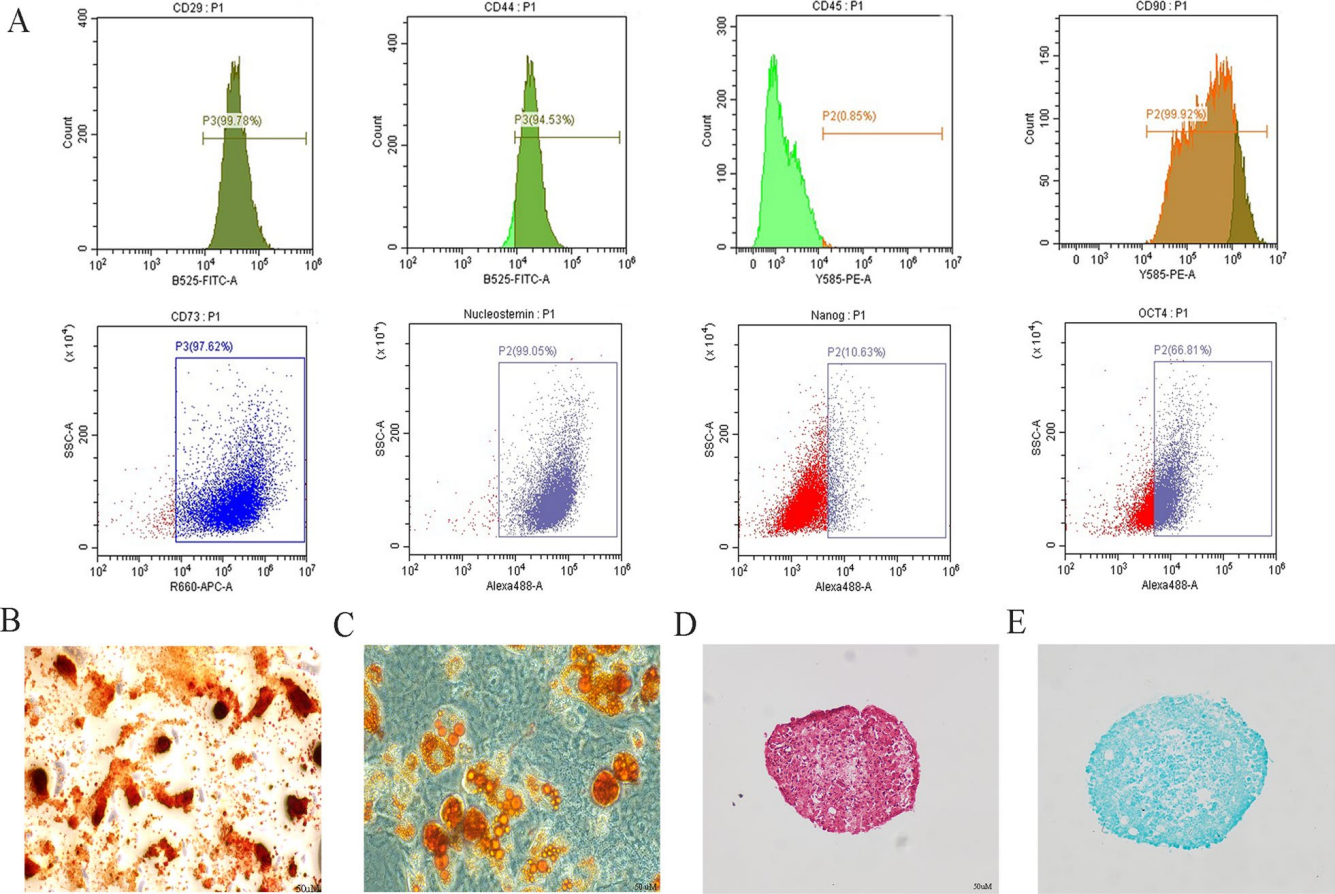
Cartilage progenitor/stem cell (CPSC) identification. The surface marker and multipotency analysis were set to confirm the stem status of CPSCs. **A** CPSCs stained with CD29, CD44, CD45 CD90, CD73, Oct-4, nanog and nucleostemin and were presented in flow cytometric assay. **B** Osteogenic differentiation of CPSCs (Alizarin Red); bar = 200 µM. **C** Adipogenic differentiation of CPSCs (Oil Red); bar = 50 µM. **D**, **E** Chondrogenic differentiation of CPSCs (Safranin O and Alcian Blue); bar = 200 µM

The stem status markers such as CD29, CD44 and CD90 and the pluripotent markers such as CD73, Oct-4, nanog and nucleostemin were detected. The results showed high levels of stem cell markers CD 29, CD44, CD 90, CD73 and nucleostemin, with more than 60% expression of Oct-4 and 10% expression of nanog in the clonogenic cells, while CD45, a leukocyte marker, was at undetectable levels (Fig. 1A). The multipotency of the clonogenic cells was detected to verify stem cell characteristics. Calcium deposition was observed in the cell layer through Alizarin Red staining (Fig. 1B). Oil Red staining showed differentiated adipocytes (Fig. 1C). Alcian blue and Safranin O staining was used to show the chondrogenic differentiation ability of CPSCs (Fig. 1D, E).

### Mitochondrial fusion is up-regulated during chondrogenic differentiation of rCPSCs, while fission is down-regulated

**Fig. 2:**
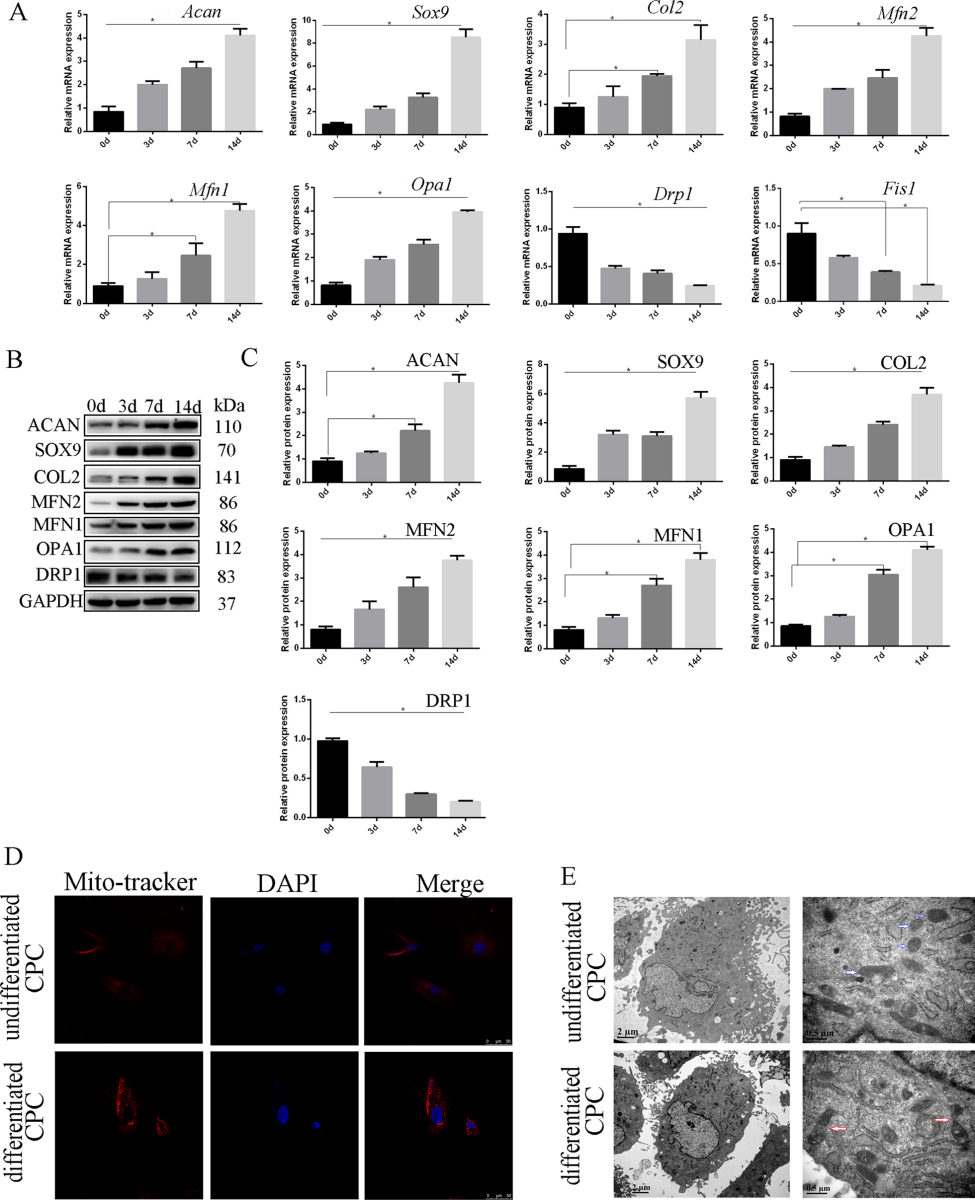
Mitochondrial fusion and fission during chondrogenic differentiation of rCPSCs. Rat CPSCs were cultured with chondrogenic induction medium for 0, 3, 7 and 14 days. **A** Chondrogenic markers and mitochondrial dynamics (fusion and fission) were measured at mRNA level. **B**, **C** Protein levels of the markers of chondrogenesis and mitochondrial dynamics (fusion and fission) were measured by western blot and quantitative analysis. **D** Mito-Tracker Red images of differentiated and undifferentiated CPSCs collected using confocal microscopy. **E** TEM images of un or -differentiated CPSC, blue arrow: normal mitochondria; red arrow: mitochondrial fusion. Bar = 2 and 0.5 µM. The data are expressed as mean ± standard deviation. *N* = 3. *P less than 0.05 versus undifferentiated CPSCs

Because cell differentiation is a process that requires enough energy, we hypothesized that changes in mitochondrial dynamics occur during commitment to differentiation. Thus, we measured markers of mitochondrial fusion and fission during the chondrogenic differentiation process of rCPSCs. Firstly, CPSCs were induced with chondrogenic induction medium, and then the chondrogenic markers such as collagen II, aggrecan, and SOX-9 were assessed to confirm that the CPSCs differentiated successfully toward chondrogenic lineage. Mitochondrial fusion markers were then evaluated to prove that fusion markers are up-regulated during the chondrogenic process of CPSCs. The levels of *Mfn2*, *Mfn1*, and *Opa*1 significantly increased during chondrogenic differentiation of rCPSCs (Fig. 2A–C), while levels of *Drp*1 and *Fis*1 decreased during chondrogenic differentiation (Fig. 2A–C). In addition, mitochondrial mass increased during chondrogenic induction compared to normal CPSCs (Fig. 2D and Additional file 1: Fig. S1A and B). Furthermore, we observed more mitochondrial fusion in the differentiated CPSC compared to undifferentiated CPSC by TEM (Fig. 2E). Our data confirmed that mitochondrial fusion and mitochondrial mass increased in commitment to chondrogenesis relative to undifferentiated CPSCs.

### The induction of mitochondrial fusion promotes differentiation of rCPSCs toward chondrogenesis

**Fig. 3:**
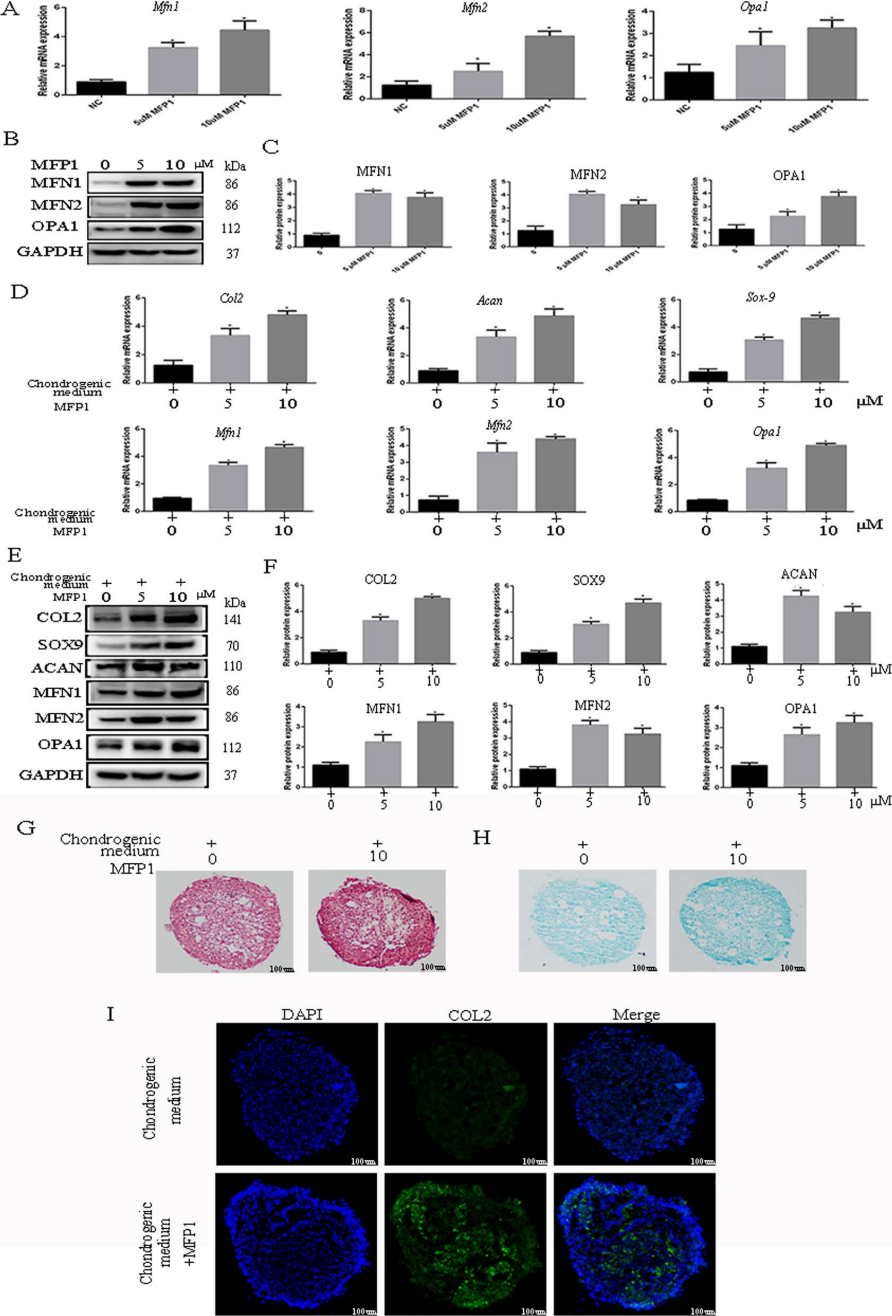
Mitochondrial fusion promoter M1 increased rCPSC differentiation toward chondrogenesis. Rat CPSCs were incubated with two different concentrations of MFP1 (5 and 10 µM). **A** The effect of MFP1 on mitochondrial fusion at mRNA level. **B**, **C** Western blot and quantitative analysis of MFP1 on mitochondrial fusion. **D**–**F** The role of MFP1 on chondrogenic and mitochondrial fusion markers at mRNA and protein levels during chondrogenic differentiation of CPSCs. **G**, **H** Safranin O and Alcian blue staining of CPSC pellet with or without MFP1. **I** *Col2* levels detected by immunofluorescence staining. DAPI (blue), *Col2* (green). Bar = 200 µM. The data are expressed as mean ± standard deviation. *N* = 3. *P less than 0.05 versus control group. *Col2* = collagen II; MFN = mitofusin; MFP1 = mitochondrial fusion promoter M1

To confirm the relationship between mitochondrial fusion and chondrogenic differentiation of CPSCs, we used mitochondrial fusion promoter 1 (MFP1) to induce mitochondrial fusion and then measured the chondrogenic markers of CPSCs. The treatment of CPSCs with MFP1 at concentrations of 5 and 10 uM are able to up-regulate levels of chondrogenic markers such as *Col2*, *Sox-9* and *Acan*. We initially used two different concentrations of MFP1 to confirm that mitochondrial fusion can be induced. The levels of *Mfn2*, *Mfn*1, and *Opa1* were measured at protein and mRNA levels. MFP1 was able to up-regulate the levels of mitochondrial fusion (Fig. 3A–C). Next, we used chondrogenic medium to induce chondrogenic differentiation of rCPSCs with or without adding MFP1. The results showed increase of chondrogenic markers at mRNA and protein levels (Fig. 3D–F). Furthermore, we found that the levels of mitochondrial fusion markers also increased in these samples. To confirm the role of MFP1 in rCPSC chondrogenic differentiation, we used Safranin O and Alcian blue staining to detect chondrogenic phenotypes. The results showed increased Safranin O and Alcian blue staining in the MFP1-treated group (Fig. 3G, H). Additionally, immunofluorescence staining showed high levels of collagen II in the MFP1-treated group relative to the non-MFP1 group (Fig. 3I).

### Mfn2 and Prg4 were co-localized in normal articular cartilage and were decreased during OA progression

**Fig. 4:**
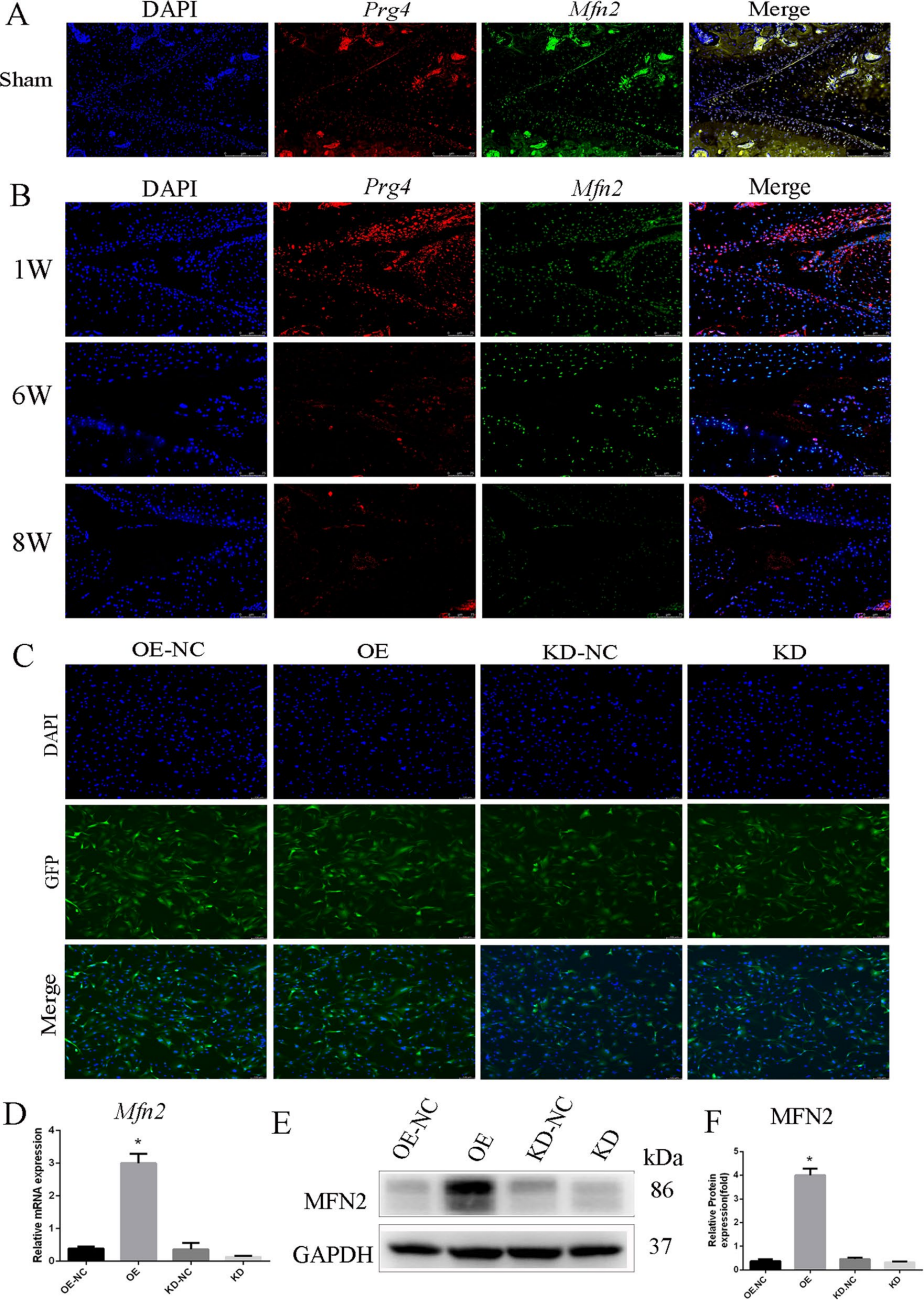
The co-localization of *Mfn2* and *Prg4* in normal cartilage. **A** The co-localization of *Mfn2* and *Prg4* in normal articular cartilage. *Mfn2* (green), *Prg4* (red), DAPI (blue). **B** Immunofluorescence staining of *Mfn2* and *Prg4* at different OA induction times (1, 6 and 8 weeks). *Mfn2* (green), *Prg4* (red), DAPI (blue). **C** rCPSCs after lentiviral transfection observed under fluorescence microscope. GFP (green), DAPI (blue). Bar = 200 µM. **D** MFN2 mRNA levels significantly up- or down-regulated compared to the negative control groups. **E**, **F** *Mfn2* protein levels significantly up- or down-regulated compared to the negative control groups, and quantitative analysis. The data are expressed as mean ± standard deviation, *N* = 3. *P less than 0.05 versus OE-NC. KD knockdown of *Mfn2*, KD-NC negative control of *Mfn2* knockdown. OE over-expression of *Mfn2*, OE-NC negative control group of *Mfn2* over-expression

Immunofluorescence (IF) double staining was done to determine the co-localization of *Mfn2* and *Prg4* in normal cartilage. Green fluorescence was conducted for *Mfn2*, while red fluorescence was conducted for *Prg4*, and the nuclei were stained with DAPI. The results showed that *Mfn2* was co-localized with *Prg4* in most cells especially on the surface layer of the cartilage (Fig. 4A). Furthermore, we induced OA by DMM in 30 (6-week-old) mice and then collected the limbs after 1, 6 and 8 weeks of the surgery. The results showed that *Mfn2* and *Prg4* gradually decreased across the different stages of OA induction (Fig. 4B).

### The transduction efficiency

We evaluated the ratio of green fluorescent protein (GFP) positive cells to the total number to quantify the efficiency of the lentiviral vectors (Fig. 4C). *Mfn2* expression was measured using qRT-PCR and western blot after 5 days of infection. mRNA and protein levels of *Mfn2* were significantly up-regulated or down-regulated relative to the control groups (Fig. 4D–F).

### Mfn2-OE increases the levels of chondrogenic genes and proteins

**Fig. 5:**
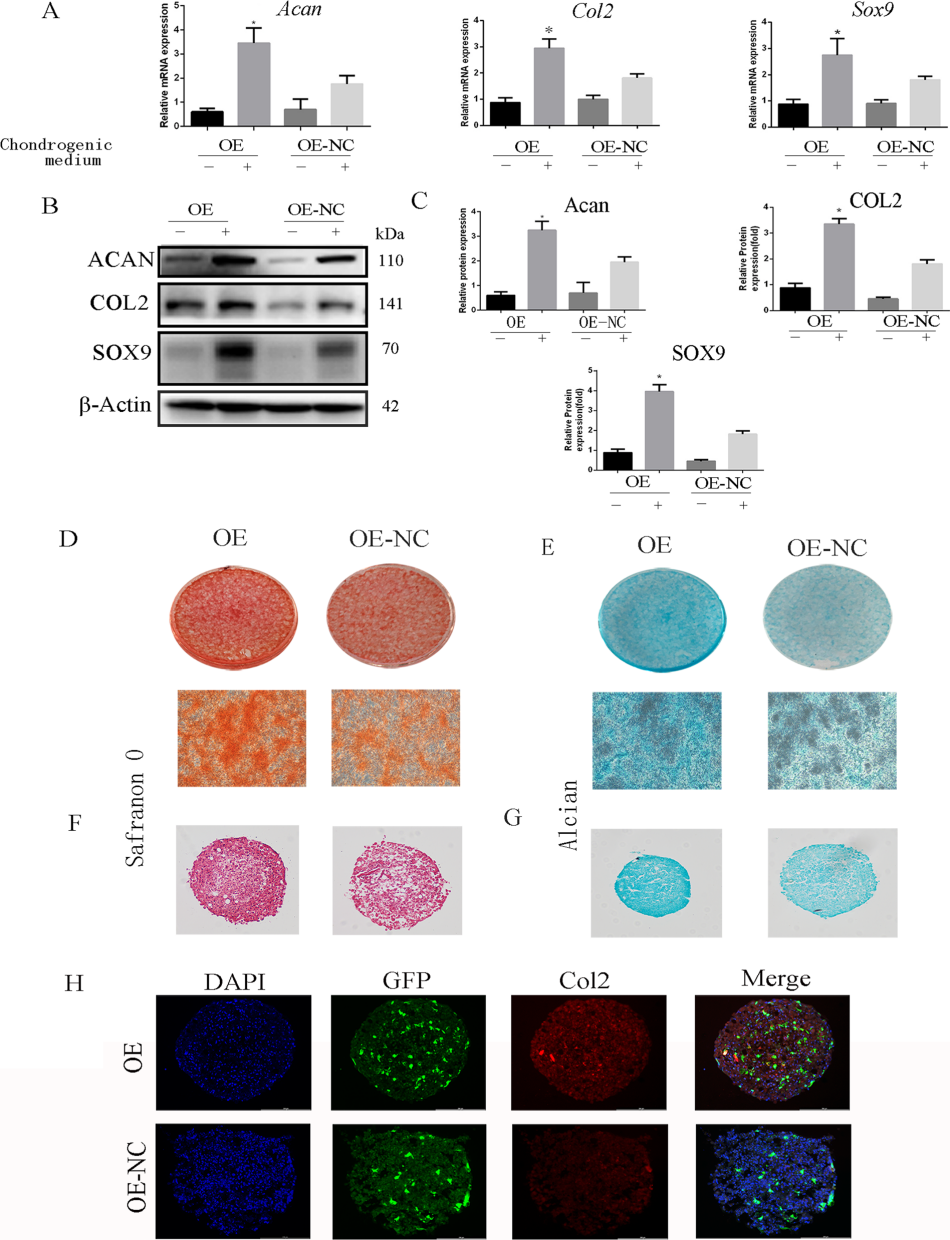
Effect of *Mfn2* on chondrogenic differentiation of CPSCs. **A** Relative mRNA expression of chondrogenic genes (*Sox-9*, *Col2*, and *Acan*) with or without chondrogenic induction medium for 14 days. **B**, **C** The expression of chondrogenic proteins (SOX-9, Col2, and Acan) with or without chondrogenic induction medium for 14 days and quantitative analysis. **D**–**G** Safranin O and Alcian blue staining of chondrogenic differentiation in plate or pellet culture with chondrogenic medium for 21 days. **H** Relative expression of Col2 (red) determined by immunofluorescence on 21 days of chondrogenesis. GFP (green), DAPI (blue). Bar = 200 µM. The data are expressed as mean ± standard deviation, *N* = 3. *P less than 0.05 versus OE-NC. OE over-expression of *Mfn2*, OE-NC negative control group of *Mfn2* over-expression

The levels of chondrogenic genes and proteins, including *Col2*, *Sox-9* and *Acan*, were detected using qRT-PCR and western blot to measure the role of *Mfn2* in differentiation of rCPSCs toward chondrogenesis. The results showed high levels of chondrogenic markers in the OE group relative to the OE-NC group (Fig. 5A, B). Furthermore, Safranin O and Alcian blue staining were used to detect glycosaminoglycan, a cartilage matrix component. The results showed that the intensity of Safranin O and Alcian blue staining increased in the OE group relative to OE-NC group (Fig. 5D–G). In addition, IF was used to detect the expression of collagen II, which increased in the OE group relative to the OE-NC group (Fig. 5H). To confirm the role of *Mfn2*-OE, we assessed the effects of OE relative to *Mfn2*-KD. The results showed higher expressions of chondrogenic markers in the OE group compare to the other groups (Additional file 2: Fig. S2A, B). No wonder, OE group has the highest intensity of Safranin O and Alcian blue staining compared to other groups (Additional file 2: Fig. S2D, E).

### Mfn2-OE reduces Notch2 signaling pathway

**Fig. 6:**
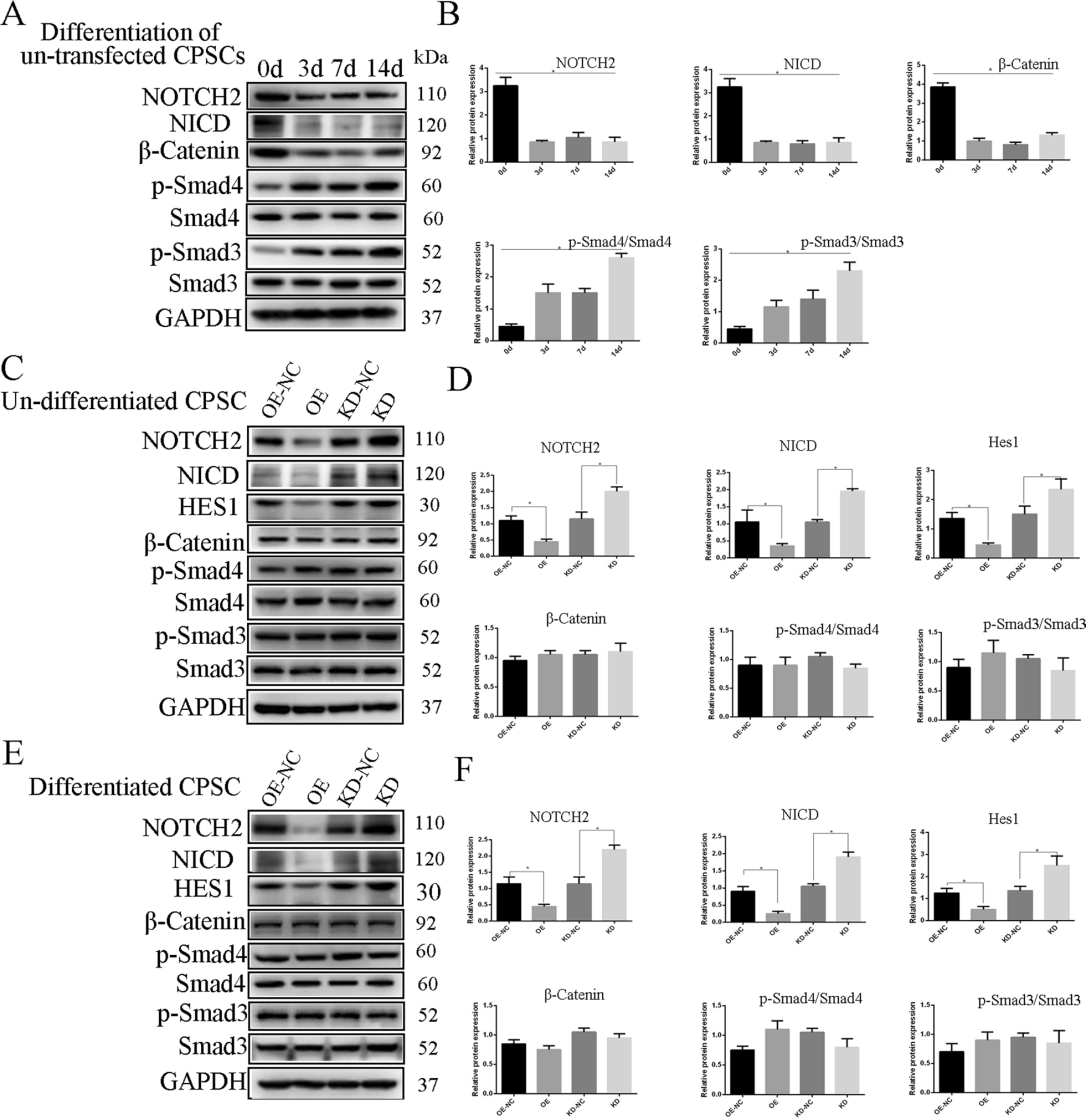
*Mfn2*- OE suppressed NOTCH2 signaling pathway. **A** Protein level expression of Notch2, smad3, smad4, and β*-catenin* on day 0, 3, 7 and 14 of chondrogenesis. **B** Relative quantitative analysis of the proteins level expression. **C**, **D** Comparison of the effects of relevant expression of Notch2, smad3, smad4, and β-catenin signaling pathways on undifferentiated CPSCs using western blot between OE-NC, OE, KD-NC, and KD group, and relative quantitative analysis. **E**, **F** Comparison of the effects of relevant expression of Notch2, smad3, smad4, and β-catenin signaling pathways on differentiated CPSCs using western blot between OE-NC, OE, KD-NC, and KD group, and relative quantitative analysis. The data are expressed as mean ± standard deviation, *N* = 3. *P less than 0.05 versus OE-NC, KD or KD-NC. OE over-expression of *Mfn2*, OE-NC negative control group of *Mfn2* over-expression, KD knockdown of *Mfn2*, KD-NC negative control of *Mfn2* knockdown

To investigate the role of Mfn2 on rCPSC chondrogenic differentiation, we investigated the signaling pathways which are involved in MSC chondrogenic differentiation, such as TGF-β (*Smad3*, *Smad4*, and *Smad2*), β*-catenin*, and Notch (*Notch1*, *Notch2*, and *Notch3*) as shown in Fig. 9. Firstly, we used chondrogenic medium to induce chondrogenesis in CPSCs at different time points. Then, western blot was run to assess the involvement of the above-mentioned signaling pathways. The results showed high levels of TGF-β signaling along with increased induction time of chondrogenic medium, while β*-catenin* and Notch signaling reduced (Fig. 6A, B). After that, we infected the cells with *Mfn2* lentiviral vectors and measured the protein levels of those signaling pathways. Interestingly, NOTCH2, NICD and HES1 were down-regulated in the OE-group (Fig. 6C, F).

### rCPSC sheets with over-expression Mfn2 ameliorates OA in vivo

**Fig. 7:**
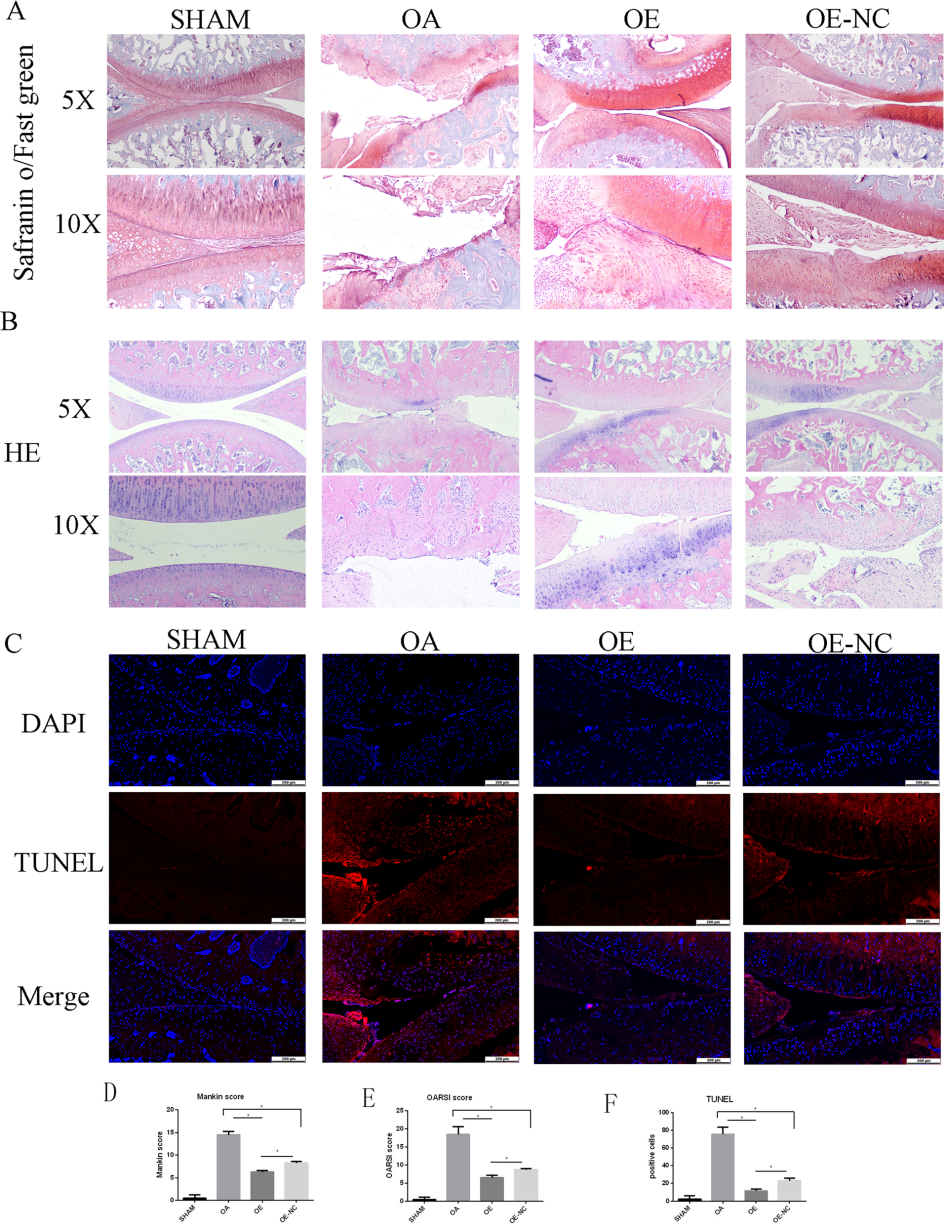
A sheet of rCPSCs with *Mfn2* over-expression ameliorated cartilage damage in a rat OA model. **A**, **B** Histological evaluation using Safranin O/ fast green and HE staining of knee joints sections from the four groups. Bar = 500 and 100 µM, 10 rats per group. **D**, **E** Modified Mankin and OARSI score system used to evaluate the four groups. **C**, **F** TUNEL staining for the cartilage of the four groups. Bar = 200 µM. *P less than 0.05. OE over-expression of *Mfn2*, OE-NC negative control group of *Mfn2* over-expression. OA = osteoarthritis, TUNEL = terminal deoxynucleotidyl transferase dUTP nick end labeling

**Fig. 8:**
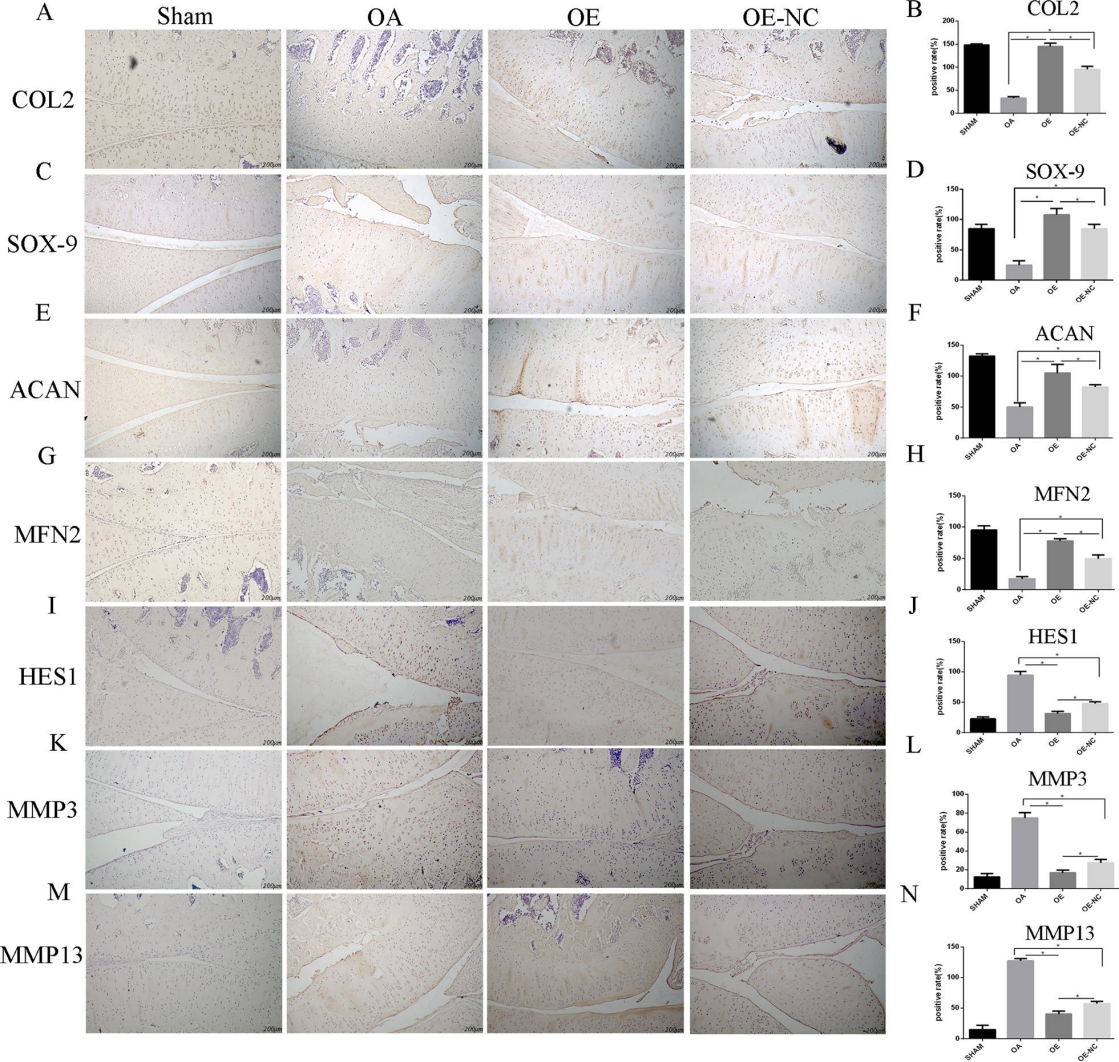
A sheet of rCPSCs with *Mfn2* over-expression increased cartilage specific proteins and decreased cartilage damage proteins in a rat OA model. **A**–**G** Immunohistochemistry staining for antibodies against Col2, SOX-9, Acan, MFN2, HES1, MMP-3 and MMP-1*3*. Bar = 200 µM. **H**–**N** Quantitative analysis of positive cells. OA = osteoarthritis, MMP = matrix metalloproteinase

To further investigate in vitro outcomes, sheets of rCPSCs with *Mfn2*-OE were used in rat OA model induced by DMM surgery. After the cells were infected with *Mfn2* len-OE and its negative control, they were injected into the knee joints of rats. In this experiment, transducted cells with OE-NC were used rather than non-transducted cells due to the comparison results between OE-NC and blank group in in vitro study. The results showed no significant difference between blank group and OE-NC group (Additional file 3: Fig. S3). Thus, we chose OE-NC as a control group for OE and OA. In addition, the control group of OE (OE-NC) has no nucleotide sequence. At eight weeks post-surgery, the histological analysis was conducted. Safranin O, hematoxylin–eosin (HE), TUNEL and immunohistochemical staining were performed. According to the Safranin O staining results, cartilage degeneration was significantly ameliorated by treatment with rCPSCs sheets compared with the OA group. Furthermore, a larger improvement was found in the OE group than in the OE-NC group (Fig. 8A). Mankin and OARSI were conducted to confirm the role of OE (Fig. 7D, E). Safranin O staining showed inhibition of synovial hyperplasia in the OE-NC group compared to the OA group, as well as greater suppression of cartilage destruction in the OE than in the OE-NC group. These results were supported by HE staining (Fig. 7B). TUNEL staining showed that the injection of rCPSCs alleviated the destructive changes and apoptosis in the cartilage matrix (Fig. 7C). Additionally, immunohistochemical study revealed that expression of chondrocyte specific markers, such as Col2, SOX-9, and Acan, increased in the OE-NC group compared to the OA group. A greater increase in the levels of these chondrocyte specific markers were observed in the OE group (Fig. 8A–C). MFN2 expression was higher in OE-group, while HES1 showed high level expression in OA group (Fig. 8D, E). OA progression markers, MMP-3 and MMP-13, were significantly reduced by the injection of rCPSCs, and lower expression levels were observed in the OE-group (Fig. 8F, G). Taken together, these results revealed that the injection of CPSCs is able to alleviate OA and showed even more promising results with over-expressed *Mfn2* cells.

## Discussion

**Fig. 9:**
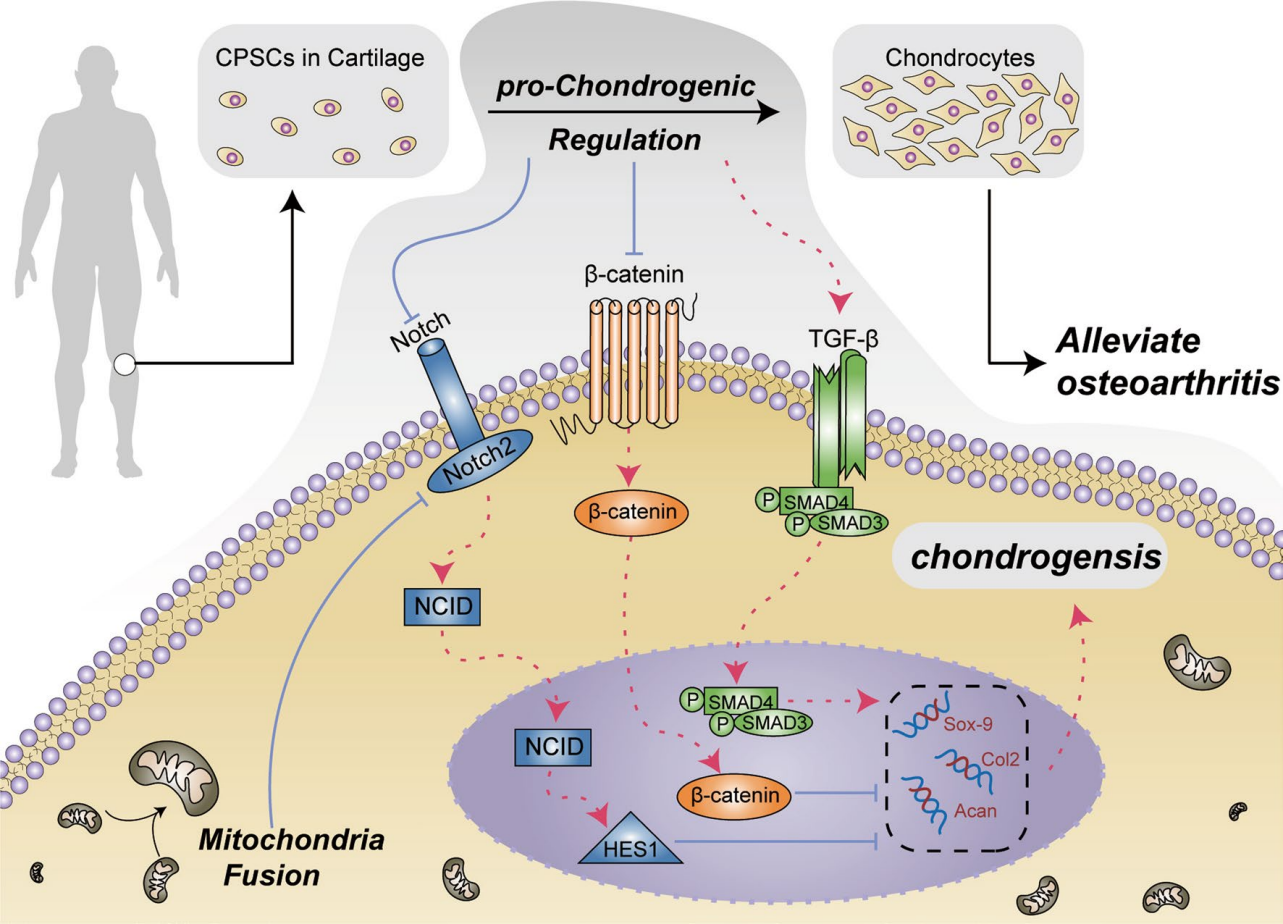
Schematic illustration of the proposed mechanisms for chondrogenic differentiation of CPSCs and the interaction of mitochondrial fusion with NOTCH2 signaling pathway

Most tissues and organs in the body have some progenitor/stem cells in a quiescent state. Their ability to self-renew and differentiate allows these cells to produce new functional cells in order to maintain the homeostasis of tissues and organs. Cartilage tissue contains a special kind of progenitor/stem cells called cartilage progenitor**/**stem cells (CPSCs). CD29, CD90 are highly expressed in these multipotent, colony forming cells [17]. Progenitor**/** stem cells produce different types of cells in a process that requires vast functional re-arrangements. Mitochondria supplement ATP which is essential for differentiation and therefore play an important role in stem cell differentiation. Previous research have reported that the shape of mitochondria changes depending on physiological states of the cell [18]. In addition, mitochondrial dynamics fission/fusion is important for MSCs differentiation, and the regulation of the dynamics can promote the differentiation process [14, 19]. However, the mechanisms by which mitochondria participate in the differentiation of CPSCs remain unclear (Fig. 9).

In the present study, we uncovered the role of mitochondrial fusion in chondrogenic differentiation of CPSCs, where promoting mitochondrial fusion leads to chondrogenic differentiation of CPSCs.

Previous studies reported that mitochondrial chemical promoter (MFP1) is able to increase levels of MFN1 and 2 [20]. After confirming the ability of MFP1 to up-regulate mitochondrial fusion, we assessed the levels of collagen II, aggrecan, and SOX-9, and found that MFP1 is able to increase chondrogenesis of CPSCs (Fig. 3). Altogether, our data demonstrate the relationship between mitochondrial fusion and chondrogenic differentiation of CPSCs, which suggest that mitochondrial fusion is able to govern differentiation of CPSCs toward chondrogenic lineage.

Mitochondrial outer and inner membrane fusion is regulated by mitofusin 1 and 2 (*Mfn1* and *Mfn2*) and optic atrophy1 (*Opa1*), respectively [21]. Knock-down of *Mfn1* and *Mfn2* resulted in fragmentation of mitochondria [22]. The fragmentation of mitochondria may indicate that a degradation process is occurring for these organelles, which is attributed to increased mitochondrial fission [14]. According to previous literature, earlier events of the chondrogenesis of MSCs could involve a fragmented phenotype of mitochondria with increasing in mitochondrial fission [19]. However, last mentioned literature showed that the expression of *Mfn2* has no significant difference between control group and chondrogenic group in the early stage of chondrogenesis, while the fission markers were increased. Our results showed that the expression of mitochondrial fusion markers increased according to the prolonged chondrogenic induction time (3, 7, 14 d). In contrast, the fission markers were decreased. Furthermore, the ETM and confocal microscopy results approved the fusion status and increasing mitochondrial mass during the chondrogenesis of CPSCs. In summary, mitochondrial fragmentation has not been observed during the chondrogenesis of CPSC due to the detection time of chondrogenesis or CPSCs and MSCs may have different performance in mitochondrial dynamic during the chondrogenic process.

Not surprisingly, loss of *Mfn2* leads to skeletal muscle atrophy and severe defects in placental trophoblastic giant cells [23]. Ablation of mitochondrial fusion proteins impairs the ability of embryonic stem cells to differentiate into cardiomyocytes [13]. Due to its vital role in mitochondrial fusion, we selected *Mfn2* to investigate the role of mitochondrial fusion in CPSC chondrogenic differentiation. The Prg4 expressing cells are mostly located on the surface and serve as progenitor cells for the deeper layers of the cartilage [24]. Thus, we evaluated the co-localization of *Mfn2* and *Prg4* in normal cartilage. The results showed that *Mfn2* and *Prg4* were co-localized in the normal cartilage (Fig. 3A). Furthermore, *Mfn2* and *Prg4* gradually decreased with extended OA induction time (Fig. 3B). From these results, we deduce that *Mfn2* may play a role in CPSC differentiation. Subsequently, we found that endogenous expression of *Mfn2* was up-regulated in rCPSCs during chondrogenesis. Therefore, we used *Mfn2*-OE or KD to promote or inhibit chondrogenesis of CPSCs and found that *Mfn2*-OE promoted chondrogenesis of rCPSCs (Fig. 5 and Additional file 2: Fig. S2).

Extensive studies have demonstrated that various signaling pathways are involved in regulating the differentiation process of progenitor cells, including Wnt, Hedgehog, Notch, and TGF-β [25]. Notch signaling pathway plays an essential role in skeletal development. Several studies have reported that Notch activation leads to suppression of chondrogenic differentiation, and over-expression of Notch1 and Notch2 in stem cells inhibit expression of chondrogenic factors [26–28]. Furthermore, the activation of Notch signaling pathway may interact with mitochondria [29]. Among these proteins, the results showed that *Mfn2*-OE is able to down-regulate *Notch2* signaling pathway (Fig. 6).

OA, is a chronic joint disease characterized by degenerative changes of the cartilage manifested as pain and limited activity of the joint. It is of great clinical significance to explore the molecular and cytological mechanisms of OA pathogenesis, in order to find new targets for prevention and treatment of OA. Various studies have reported the relationship between OA and mitochondrial dysfunction [30, 31]. It has been reported that the Notch pathway is remarkably up-regulated during OA development [32].

To further investigate the findings in vitro, rCPSC sheets with *Mfn2*-OE or OE-NC were injected intra-articulary in the rat OA model. The cartilage was assessed in the animal experiment, and erosion and loss of cartilage matrix was observed in the OA group. The cartilage damage was ameliorated by the injection of CPSCs with *Mfn2*-OE negative control and more improvement was observed in the OE group (Fig. 7). Immunohistochemistry staining showed that levels of chondrogenic markers, such as Col2, SOX-9 and Acan, increased in the OE-NC group and even more so in the OE group compared to the OA group (Fig. 8). Furthermore, *Mfn2*-OE ameliorated OA according to the OARSI and Mankin score, and reduced extracellular matrix destruction markers, such as MMP-3 and MMP-13 according to immunohistochemistry staining (Figs. 7 and 8).

## Conclusion

The present study corroborates the role of mitochondrial fusion in chondrogenic differentiation of CPSCs via regulating the *Notch2* signal pathway. All of the findings of the in vitro experiments were taken seriously and applied in vivo. Our findings suggest that mitochondrial fusion accelerates chondrogenic differentiation of CPSCs and holds therapeutic potential for the treatment of OA.

## Supplementary Information

**Additional file 1: Fig. S1.** Mito-Tracker Red images of differentiated and undifferentiated CPSCs. **A** CPSCs were seeded in 24-well plate and then incubated with or without chondrogenic medium for 14 d. The images were captured with immunofluorescence microscopy. Mito-Tracker Red (Red), DAPI (blue). **B** The quantitative analysis.

**Additional file 2: Fig. S2.** The effect of MFN2 OE and KD on CPSC chondrogenic differentiation. **A** Relative mRNA expression of chondrogenic genes (*Sox-9*, *Col2*, and *Acan*) at day 14 of chondrogenesis. **B**, **C** The expression of chondrogenic proteins (SOX-9, Col2, and Acan) at day 14 of chondrogenesis, and quantitative analysis. **D**, **E** Safranin O and Alcian blue staining of chondrogenic differentiation in plate culture with chondrogenic medium for 21 days. Bar = 200 µM. The data are expressed as mean ± standard deviation, *N* = 3. *P less than 0.05 versus OE-NC, KD or KD-NC. OE over-expression of *Mfn2*, OE-NC negative control group of *Mfn2* over-expression, KD knockdown of *Mfn2*, KD-NC negative control of *Mfn2* knockdown.

**Additional file 3: Fig. S3.** The comparison between blank group and OE-NC group. **A** Cell counting Kit-8 was used to compare between the two groups at 24 h and 48 h. **B** The expression of chondrogenic markers of CPSCs at mRNA level using qRT-PCR. **C** Western blot was used to evaluate the chondrogenic markers of CPSCs at protein level. The data are expressed as mean ± standard deviation, *N* = 3. *P less than 0.05 versus OE-NC. OE-NC negative control group of *Mfn2* over-expression.

## Data Availability

The data in this study are available from the corresponding author on reasonable request.

## Abbreviations

MSCs: Mesenchymal stem @cells
rCPSC: Rat cartilage progenitor**/**stem cells
OA: Osteoarthritis
TEM: Transmission electron microscopy
*Mfn2*: Mitofusin 2
DRP1: Dynamic-related protein
*Mfn1*: Mitofusin 1
*Fis1*: Fission
*Acan*: Aggrecan
FBS: Fetal bovine serum
MMP: Matrix metalloproteinase
DMEM: Dulbecco’s modified eagle medium
qRT-PCR: Quantitative RT-PCR
GAPDH: Glyceraldehyde-3-phosphate dehydrogenase
PBS: Phosphate-buffered saline
NC: Negative control
OE: Overexpress
DMM: Destabilization of the medial meniscus
Opa1: Optic atrophy1
IF: Immunofluorescence
HE: Hematoxylin–eosin
TUNEL: Terminal deoxynucleotidyl transferase dUTP nick end labeling
TGF-β: Transforming growth factor-β
