# Supplementary figures and images for: The effect of mitochondrial fusion on chondrogenic differentiation of cartilage progenitor/stem cells via Notch2 signal pathway
Source: Stem Cell Res Ther. 2022 Mar 25;13:127. doi: 10.1186/s13287-022-02758-7 (PMC8951683; doi:10.1186/s13287-022-02758-7)

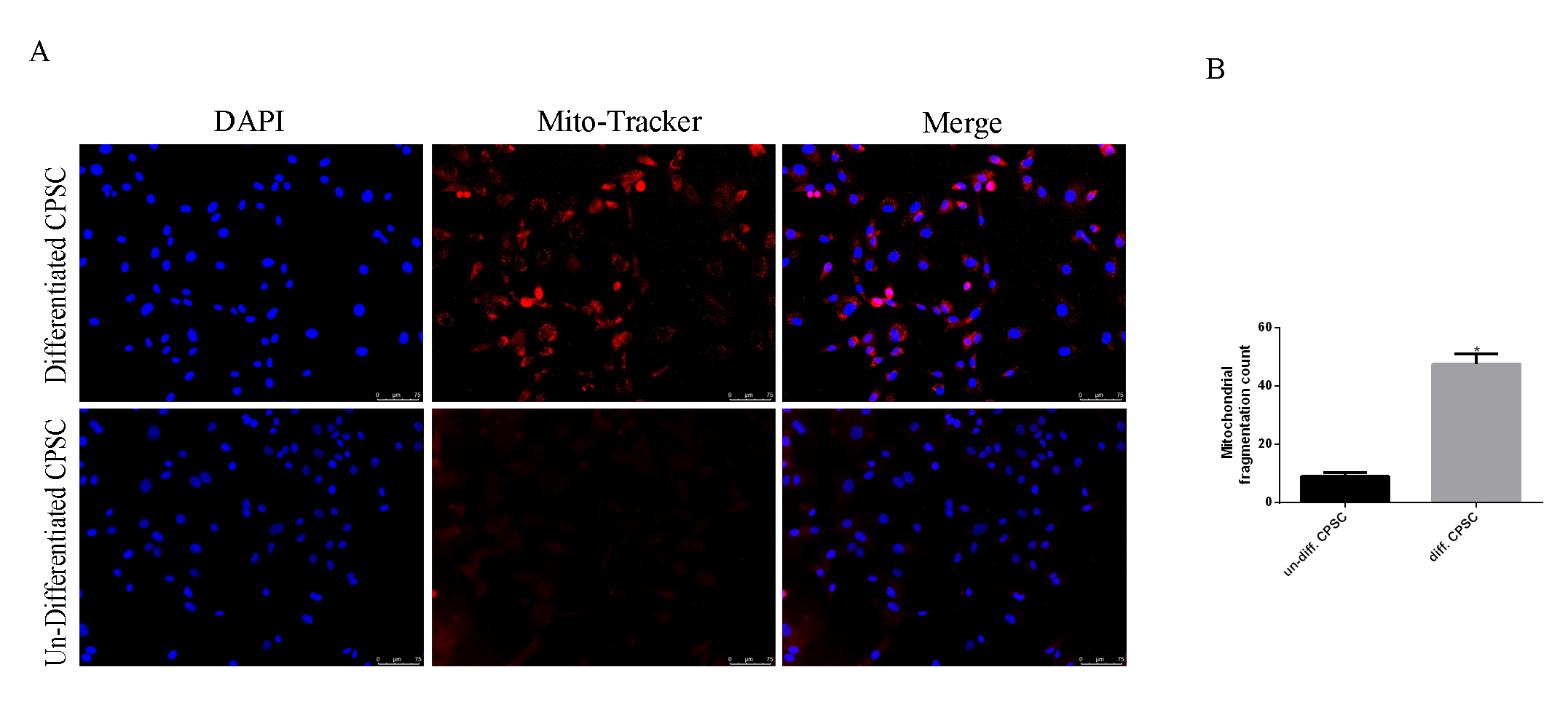

Supplement: Supplementary file 1 — Additional file 1: Fig. S1. Mito-Tracker Red images of differentiated and undifferentiated CPSCs. A CPSCs were seeded in 24-well plate and then incubated with or without chondrogenic medium for 14 d. The images were captured with immunofluorescence microscopy. Mito-Tracker Red (Red), DAPI (blue). B The quantitative analysis. [file 13287_2022_2758_MOESM1_ESM.tif]

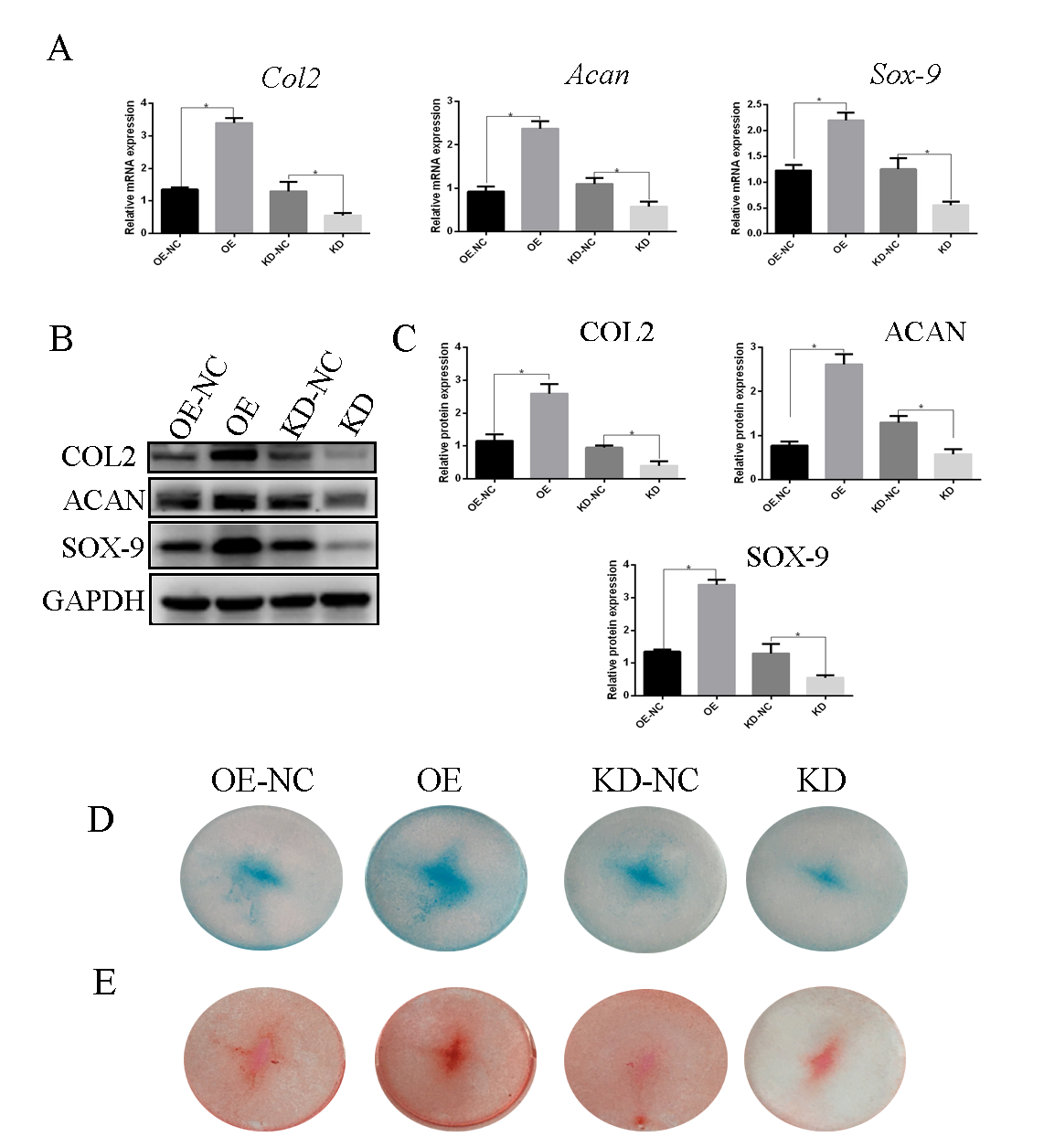

Supplement: Supplementary file 2 — Additional file 2: Fig. S2. The effect of MFN2 OE and KD on CPSC chondrogenic differentiation. A Relative mRNA expression of chondrogenic genes (Sox-9, Col2, and Acan) at day 14 of chondrogenesis. B, C The expression of chondrogenic proteins (SOX-9, Col2, and Acan) at day 14 of chondrogenesis, and quantitative analysis. D, E Safranin O and Alcian blue staining of chondrogenic differentiation in plate culture with chondrogenic medium for 21 days. Bar = 200 µM. The data are expressed as mean ± standard deviation, N = 3. *P less than 0.05 versus OE-NC, KD or KD-NC. OE over-expression of Mfn2, OE-NC negative control group of Mfn2 over-expression, KD knockdown of Mfn2, KD-NC negative control of Mfn2 knockdown. [file 13287_2022_2758_MOESM2_ESM.tif]

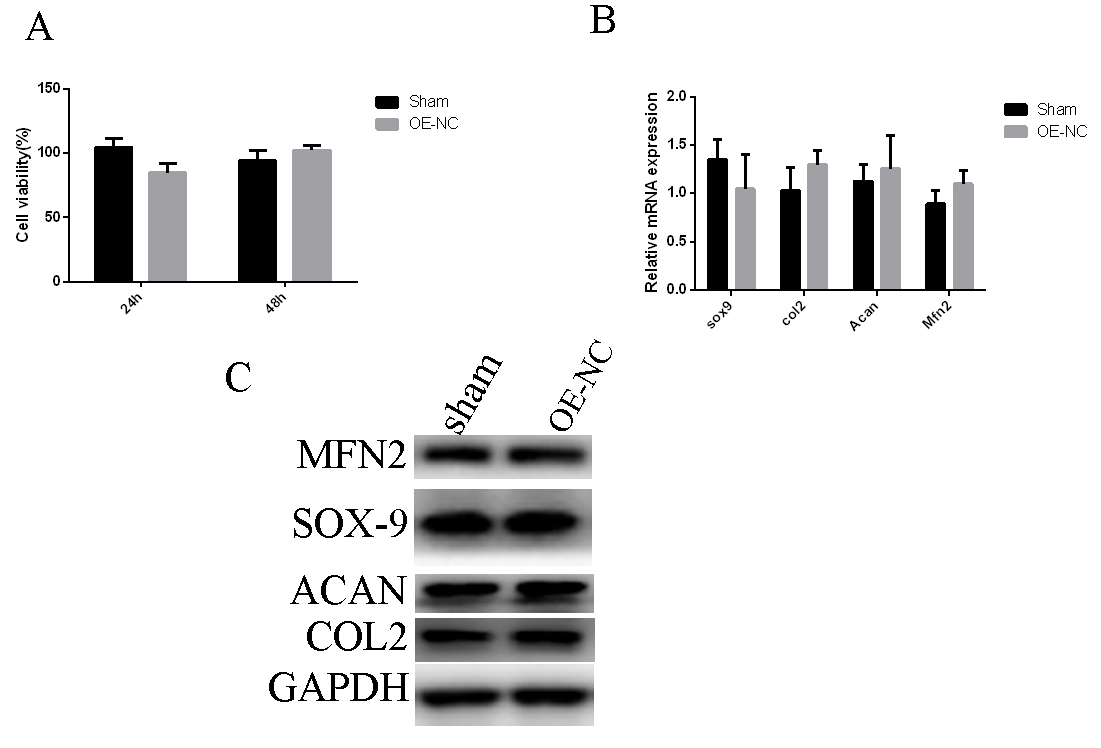

Supplement: Supplementary file 3 — Additional file 3: Fig. S3. The comparison between blank group and OE-NC group. A Cell counting Kit-8 was used to compare between the two groups at 24 h and 48 h. B The expression of chondrogenic markers of CPSCs at mRNA level using qRT-PCR. C Western blot was used to evaluate the chondrogenic markers of CPSCs at protein level. The data are expressed as mean ± standard deviation, N = 3. *P less than 0.05 versus OE-NC. OE-NC negative control group of Mfn2 over-expression. [file 13287_2022_2758_MOESM3_ESM.tif]
